# Scapulohumeral kinematics and neuromuscular control during scaption are associated with passive stiffness and strength of periscapular muscles in competitive adolescent swimmers

**DOI:** 10.1038/s41598-023-27920-w

**Published:** 2023-01-13

**Authors:** Po-Tsun Chen, Po-Kai Wang, Ting-Ting Chen, Ho-Yi Tuan-Mu, Chih-Hao Chiu, Kuan-Lin Liu

**Affiliations:** 1 School of Physical Therapy, Chang Gung University, Taoyuan, Taiwan; 2 Department of Orthopedic Surgery, Chang Gung Memorial Hospital, Taoyuan, Taiwan; 3 Department of Anesthesiology, Hualien Tzu Chi Hospital, Buddhist Tzu Chi Medical Foundation, Hualien, Taiwan; 4 Sports Medicine Center, Hualien Tzu Chi Hospital, Buddhist Tzu Chi Medical Foundation, Hualien, Taiwan; 5 School of Medicine, Tzu Chi University, Hualien, Taiwan; 6 Department of Leisure Industry and Health Promotion, National Ilan University, Yilan, Taiwan; 7 Department of Physical Therapy, Tzu Chi University, Hualien, Taiwan; 8 Department of Orthopedics, Hualien Tzu Chi Hospital, Buddhist Tzu Chi Medical Foundation, Hualien, Taiwan

**Keywords:** Skeletal muscle, Orthopaedics

## Abstract

The passive stiffness and strength of periscapular muscles may affect scapulohumeral control, especially in overhead athletes due to sports-specific training. This study tried to assess the relationship between the passive stiffness and strength of periscapular muscles, scapulohumeral kinematics and neuromuscular control during scaption in swimmers. Ten male adolescent competitive front-crawl swimmers were recruited. The passive stiffness and strength of periscapular muscles were measured in standard postures by a hand-held myotonometer and dynamometer, respectively. Surface electromyography and electromagnetic tracking systems were synchronized to record the muscle activities and scapulohumeral kinematics during scaption. Correlations between the passive stiffness or strength of periscapular muscles and the kinematics or muscle activity were examined by Spearman's rank correlation coefficient. The maximal strength of periscapular muscles correlated positively with the ranges of upward and external rotation of the scapula and negatively with muscle activity during scaption. Passive stiffness of periscapular muscles was associated with the downward rotation of the scapula and triggered an increase in muscle activity. Increased passive stiffness or decreased strength in the periscapular muscles may affect their role in controlling the scapular rotation and contribute to compensation from adjacent muscles. Our findings suggest that when attempting to evaluate scapular behavior, it may be beneficial to examine muscle strength and passive stiffness of periscapular muscles.

## Introduction

Swimmers are frequently molested by shoulder pain. This symptom is commonly referred to as the swimmer’s shoulder and may cause a decline in performance or even end a swimmer’s career. The prevalence of shoulder pain in the front-crawl style (52.44%) is higher than in other stroke types^1^ Pain was reported as especially noticeable during the early-pull-through phase of the front-crawl style, which is regarded as the posture inducing the subacromial impingement syndrome^1,2^.

The scapula provides a stable support for arm movements during swimming. Once scapula dyskinesis (SD) appears in athletes, the risk of developing shoulder pain is increased by 43%^3^ SD is commonly seen in the non-breathing side^4^ or the injured shoulder^5^ of competitive front-crawl swimmers. During scaption, internal rotation and anterior tilting of the scapula are more pronounced in young competitive swimmers than in non-practitioners^6^ Since SD is connected to abnormalities in neuromuscular control related to weakness or stiffness of periscapular muscles^7–10^ muscle activity should be comprehensively evaluated within training programs.

As mentioned, SD may be accompanied by a decline in the strength of periscapular muscle. Overhead athletes with SD were found to have reduced strength of the lower trapezius (LT) compared to athletes without SD^11^ This effect is possibly caused by sport-specific training for competitive swimmers focusing on technique, which may ignore the strength balance between muscles. Long term, the strength of the LT in teenage elite swimmers decreases after three years of swim training, whereas the strength of other shoulder muscles increases^12^ Unbalanced periscapular muscle strength may disrupt the function of force coupling on the scapular rotations in overhead athletes. Approximately 85% of swimmers with asymptomatic shoulders have altered scapula movement immediately after a single swim training session^8^ When compared to non-swimmers, elite swimmers seem to have more scapular protraction during scaption^13^ However, it remains controversial whether SD is the cause or the result of symptomatic shoulders. Middle trapezius (MT) strength in swimmers with symptomatic shoulders was found to be lower than in swimmers with asymptomatic shoulders, even though no difference in SD seems to occurr between groups^14^.

Muscle passive stiffness commonly appears after sports training and restricts the active range of movement. It was found that the range of shoulder external rotation was reduced after a two-hour swim training session due to the repetitive force production of the shoulder internal rotator muscles^15^ The stiffness of the shoulder rotator muscles affects not only how flexible the shoulder is, but also how the muscles control the movement of the scapulohumeral joint. One study showed that swimmers had an increase in external rotation and posterior tilt of the scapula during arm elevation after reducing pectoralis minor stiffness through manual stretching^16^ In another study, although the muscle does not insert directly into the scapula, the latissimus dorsi stiffness was associated with an increased upward rotation and decreased posterior tilt of the scapula during scaption in swimmers^9^ It is still uncertain whether the stiffness of periscapular muscles is associated with the neuromuscular controls of scapulohumeral kinematics in swimmers.

Therefore, this study aimed to investigate the relationship between the stiffness and strength of periscapular muscles and the neuromuscular control of scapulohumeral movement in competitive adolescent swimmers. The passive stiffness and maximal strength were evaluated in periscapular muscles, and the overall shoulder kinematics and the neuromuscular control of periscapular muscles during scaption were measured.

## Methods

### Participants

For this correlational study, ten male competitive adolescent swimmers (age: 16.5 ± 0.6 years, height: 170.5 ± 10.1 cm, weight: 60.6 ± 10.3 kg) from a high school team were recruited. All recruited swimmers had practiced middle- (200 and 400 m) and long- (800 and 1500 m) distances and participated in swimming competitions for at least two years. The front-crawl style was their main stroke pattern during training and competitions. They received swim training for three hours per day and five days per week. To eliminate other symptoms that could affect shoulder neuromuscular control, the author (P.-T. C.), who was a physical therapist with nineteen years of experience, performed physical examinations to rule out shoulder disorders (i.e., rotator cuff tendinopathy, subacromial impingement syndrome, glenohumeral joint instability, cervical radiculopathy, or thoracic outlet syndrome). Swimmers were excluded if any musculoskeletal or neurological symptoms on the upper extremity were noticed. After explanation of study purpose and procedures, the participants signed the informed consent before enrolment. This study was conducted in accordance with the Declaration of Helsinki and approved by the review board of Hualien Tzu Chi Hospital.

### Procedures and instruments

#### Procedure

After ensuring the eligibility of participants, basic information (height and weight, age, breathing side during swimming, etc.) was obtained. The passive stiffness and strength of periscapular muscles and the neuromuscular control of scapulohumeral kinematics of the participant’s non-breathing shoulder were measured. To avoid immediate soreness after muscle exertion, passive stiffness measurements were performed first, and the maximal strength of the periscapular muscles was determined at the end of the experiment. Following the stiffness measurement, the scapulohumeral kinematics and the muscle electromyographic activity of periscapular muscles during scaption were recorded. Finally, the maximal strength of the periscapular muscles was tested.

#### Muscle passive stiffness

Participants were asked to lie prone on a treatment table with arms by the side in a relaxed position for measurements. A hand-held myotonometer (MyotonPRO, Myoton AS, Estonia) was used to measure the muscle passive stiffness (N/m) by placing the sensing probe on the upper trapezius (UT), middle trapezius (MT), LT and serratus anterior (SA). The measuring location of each muscle was determined as the following: UT, a point 2 cm lateral to the midpoint between the spinous process of the 7th cervical vertebra and the lateral edge of the acromion^17^; MT, the midpoint between the spinous process of the 2nd thoracic vertebra and the medial edge of scapula; LT, the midpoint of a line inclined at 55° from the point 5 cm below the root of spine of scapula down to the intersection with the spinous process of thoracic vertebrae; and SA, the midaxillary line at the level of the inferior angle of the scapula along the rib^18^ The mean passive stiffness from three trials was obtained.

### Scapulohumeral kinematics

For the kinematics measurement, three receivers of an electromagnetic motion tracking system (FASTRAK, Polhemus, USA) were attached to the sternum notch, lateral plateau of acromial process, and lateral side of distal humerus to record the motion of the thorax, scapula and humerus, respectively. Participants were asked to sit erect in front of the electromagnetic transmitter to minimize the effects of spine posture^19^ and the height was adjusted at the level of each participant’s shoulder joint. Then, ten bony landmarks recommended by the International Society of Biomechanics^20^ were captured by the digitizer in synchrony with the receiver’s position and orientation to construct the local coordinate system (LCS) of each segment. The bony landmarks consisted of incisura jugularis, processus xiphoideus, the spinous process of the 7th cervical vertebra, the spinous process of the 8th thoracic vertebra, trigonum scapulae, angulus inferior of scapulae, angulus acromialis, processus coracoideus, and lateral and medial epicondyles of humerus. After digitization, the participants performed scaption at a comfortable speed (two seconds for elevation and two seconds for lowering) over five trials. A color tape was applied to the ground (a projection line inclined at 30° anterior to the frontal plane) as the guiding mark of the scapular plane for the scaption movement. The kinematic data were collected at a sampling rate of 120 Hz and synchronized with the surface electromyography.

### Surface electromyography (sEMG)

A wireless sEMG (Trigno, Delsys, USA) was utilized to record the muscle activities of UT, MT, LT, SA and middle fiber of the deltoid (MD) during scaption and maximal muscle strength measurements. The surface skin of each muscle was prepared and cleaned with an alcoholic swab and hair shaving. The location for electrode placement of each muscle was palpated and identified: UT, halfway between the spinous process of the 7th cervical vertebra and the acromion; MT, halfway on the horizontal line between the thoracic spine and the root of the scapular spine; LT, obliquely upward and laterally along a line between the intersection of the scapular spine with the vertebral border of the scapula, and the spinous process of 7th thoracic vertebra; SA, anterior to the latissimus dorsi and posterior to the pectoralis major; IS, approximately 4 cm below the spine of the scapula on the lateral aspect over the infrascapular fossa; and MD, on the lateral aspect of the upper arm and approximately 3 cm below the acromion^21^. The data of sEMG was sampled at a frequency of 1000 Hz.

### Maximal muscle strength

The strength of UT, MT, LT, SA and MD during maximal voluntary isometric contraction (MVIC) were assessed using a handheld dynamometer (microFET3, Hoggan Scientific, USA). The posture and resistance for each muscle assessment were according to previous studies^22,23^. For UT, while sitting upright with head in neutral and arm by the side, resistance was applied to the superior aspect of the acromial plateau. For MT, with shoulder abduction at 90° with thumb toward the ceiling in a prone position, resistance was given on the lateral aspect of the spine of the scapula. For LT, with shoulder abduction at 145° with thumb toward the ceiling in a prone lying posture, resistance was applied at the same location as for MT. For SA, with both shoulder and elbow flexion at 90° with shoulder internal rotation in a supine position, resistance was applied on the proximal site of the ulnar near olecranon. For MD, while sitting upright with the shoulder in neutral and arm by the side, resistance was given on the lateral side of the distal humerus. Participants were asked to perform two trials of five-second MVICs against the dynamometer for each muscle. For the subsequent normalization processing, the electromyographic activity of each muscle was recorded during the respective MVIC measurement.

### Data reduction

**Figure 1:**
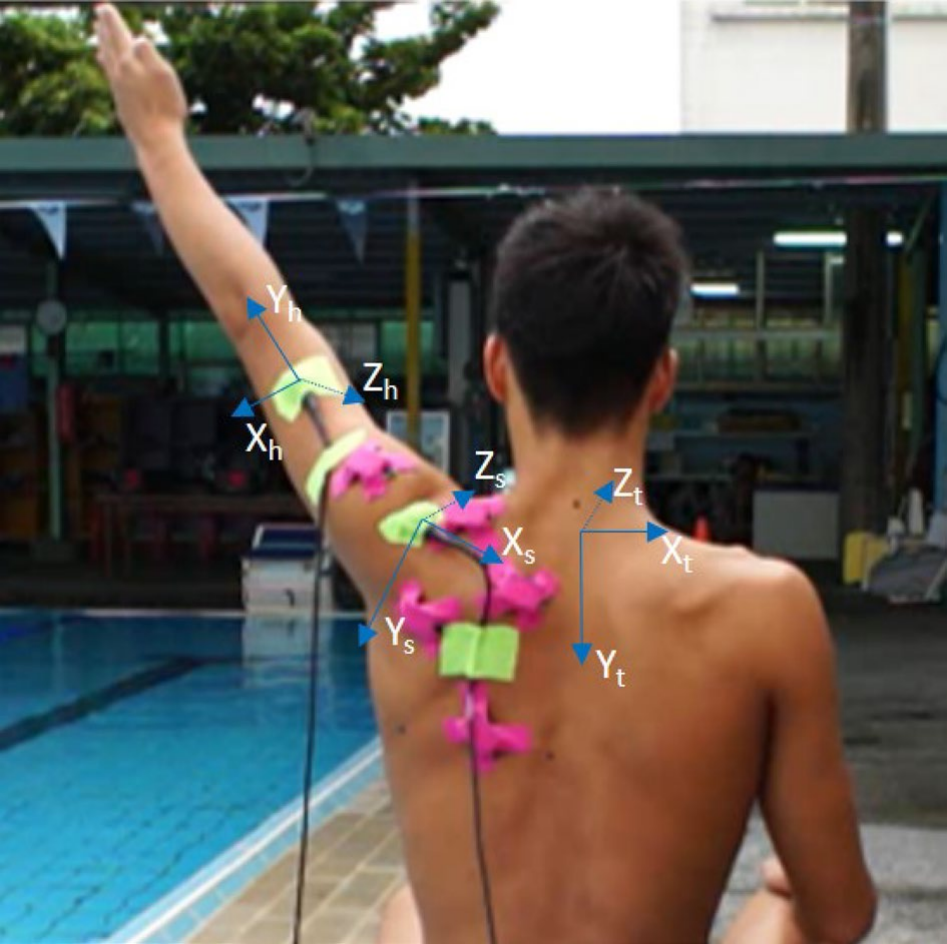
The swimmer’s left shoulder as the non-breathing side during swimming was evaluated. The LCS of thoracic ([X_t_, Y_t_, Z_t_]), humerus ([X_h_, Y_h_, Z_h_]) and scapula ([X_s_, Y_s_, Z_s_]). The X axis is pointing towards the right, the Y axis inferiorly, and the Z axis anteriorly.

The spatial relationship between the position of bony landmarks and the orientation of the receiver on the same segment in static status was used to derive virtual bony landmarks during scaption. The LCS of each segment was determined by the virtual landmarks for every time point, where the Y axis was in the inferior-superior direction, the X axis was in the posterior-anterior direction and the Z axis was in the medial–lateral direction (Fig. 1)^20^. By transforming the LCS of the distal segment to the LCS of the proximal segment, the rotational matrix of humerus-thorax (HT) and scapula-thorax (ST) can be determined. Then, the three-dimensional HT and ST movement can be obtained from the rotational matrices by the Euler sequences Y-X’-Y’’ and Y-X’-Z’’, respectively^40^. Finally, the angle of abduction (+ / − , arm elevation/lowering) of HT and the internal rotation (+ / − , internal/external rotation), downward rotation (+ / − , downward/upward rotation) and posterior tilting (+ / − , posterior/anterior tilting) of ST were calculated.

All raw sEMG signals recorded during scaption and MVIC were rectified and filtered with a band-pass filter of 20–500 Hz (4th order Butterworth). Then the signal was smoothed by the root mean square value with a 50 ms moving window. The electromyographic activity of each muscle during scaption was normalized to the peak value during the MVIC test (%MVIC).

### Data analysis

Scapular kinematics was analyzed within the range of scaption 0° to 120° suggested by the validity study^24^ The rotation angles of the ST movement were extracted at every 30° of HT elevation (ele_30°_, ele_60°_, ele_90°_ and ele_120°_) and lowering phases (low_120°_, low_90°_, low_60°_ and low_30°_) of scaption. Each muscle activity was averaged for 30° increments of the scaption, using intervals of 0–30° (ele_0–30°_), 30–60° (ele_30–60°_), 60–90° (ele_60–90°_) and 90–120° (ele_90–120°_) of HT elevation, and of 120–90° (low_120–90°_), 90–60° (low_90–60°_), 60–30° (low_60–30°_) and 30–0° (low_30–0°_) lowering.

The relationships between muscle stiffness and strength with regard to the scapulohumeral kinematics and muscle activity were examined by Spearman's rank correlation coefficient (r_s_), and the significance level was set at 0.05.

## Results

### Relationship between muscle passive stiffness and scapula kinematics

The passive stiffness of UT positively correlated with the downward rotation of the scapula at ele_60°_ (r_s_ = 0.685, *p* = 0.029) and ele_90°_ (r_s_ = 0.661, *p* = 0.038) (Table 1). The passive stiffness of SA negatively correlated with the downward rotations at low_120°_ (r_s_ =  − 0.661, *p* = 0.038), low_90°_ (r_s_ =  − 0.891, *p* = 0.001) and low_60°_ (r_s_ =  − 0.648, *p* = 0.043) (Table 1). The passive stiffness of MT positively correlated with the internal rotation of the scapula at low_90°_ (r_s_ = 0.648, *p* = 0.043) and low_60°_ (r_s_ = 0.673, *p* = 0.033) and negatively correlated with the posterior tilting of the scapula at low_120°_ (r_s_ =  − 0.758, *p* = 0.011) (Table 1).

**Table 1:** The relationship between the passive stiffness of scapular muscles and the scapular kinematics.

Scapula kinematics		Muscle passive stiffness							
		UT		MT		LT		SA	
		r_s_	*p*	r_s_	*p*	r_s_	*p*	r_s_	*p*
Internal rotation	ele_30°_	− .030	.934	.370	.293	− .128	.725	.115	.751
	ele_60°_	− .103	.777	.515	.128	− .213	.555	− .006	.987
	ele_90°_	− .503	.138	.527	.117	− .535	.111	.079	.829
	ele_120°_	− .588	.074	.382	.276	− .620	.056	.042	.907
	low_120°_	− .091	.803	.552	.098	− .128	.725	− .176	.627
	low_90°_	− .394	.260	.673*	**.033**	− .419	.228	− .139	.701
	low_60°_	− .600	.067	.648*	**.043**	− .523	.121	.030	.934
	low_30°_	− .709*	**.022**	.394	.260	− .602	.066	.018	.960
Downward rotation	ele_30°_	.600	.067	.188	.603	.419	.228	.042	.907
	ele_60°_	.685*	**.029**	.079	.829	.468	.172	− .006	.987
	ele_90°_	.661*	**.038**	− .030	.934	.395	.258	− .139	.701
	ele_120°_	.442	.200	− .370	.293	.213	.555	− .539	.108
	low_120°_	.467	.174	.115	.751	.085	.815	− .455	.187
	low_90°_	.467	.174	.067	.855	.073	.841	− .648*	**.043**
	low_60°_	.152	.676	− .152	.676	− .195	.590	− .891*	**.001**
	low_30°_	.248	.489	− .345	.328	.170	.638	− .661*	**.038**
Posterior tilting	ele_30°_	.479	.162	− .539	.108	.304	.393	.370	.293
	ele_60°_	.503	.138	− .479	.162	.292	.413	.333	.347
	ele_90°_	.588	.074	− .564	.090	.322	.364	.248	.489
	ele_120°_	.455	.187	− .624	.054	.219	.544	.139	.701
	low_120°_	− .127	.726	− .321	.365	.292	.413	.358	.310
	low_90°_	.212	.556	− .564	.090	.255	.476	.297	.405
	low_60°_	.406	.244	− .576	.082	.255	.476	.139	.701
	low_30°_	.455	.187	− .758*	**.011**	.261	.466	− .055	.881

*Significant correlation between the muscle passive stiffness and the scapular kinematics (*p* < 0.05).

*P* values set in bold indicate statistical significance.

### Relationship between muscle passive stiffness and muscle activity

There was no correlation between the possive stiffness of the periscapular muscle in resting status with its own sEMG activity during scaption. However, the muscle passive stiffness positively correlated with the activity of other adjacent muscles, so the passive stiffness of LT positively correlated with the sEMG of SA (ele_0–30°_, ele_60–90°_, ele_90–120°_, low_120–90°_,low_90–60°_, r_s_ = 0.723, *p* = 0.018; low_60–30°_, r_s_ = 0.665, *p* = 0.036; low_30–0°_, r_s_ = 0.904, *p* < 0.001) (Table 2) and the passive stiffness of SA also positively correlated with the sEMG of UT (low_120–90°_, r_s_ = 0.758, *p* = 0.011) (Table 2).

**Table 2:** The relationship between the muscle passive stiffness and the muscle activity of periscapular muscles.

Muscle activity		Muscle passive stiffness							
		UT		MT		LT		SA	
		r_s_	*p*	r_s_	*p*	r_s_	*p*	r_s_	*p*
UT	ele_0-30°_	− .091	.803	.115	.751	.067	.854	.333	.347
	ele_30–60°_	.103	.777	.067	.855	.146	.688	.358	.310
	ele_60–90°_	.297	.405	.067	.855	.359	.309	.273	.446
	ele_90–120°_	.236	.511	.442	.200	.243	.498	.491	.150
	low_120–90°_	− .018	.960	.503	.138	.456	.185	.758*	**.011**
	low_90–60°_	− .152	.676	.636*	**.048**	.079	.828	.430	.214
	low_60–30°_	− .552	.098	.612	.060	− .255	.476	.018	.960
	low_30–0°_	− .564	.090	.418	.229	− .109	.763	.067	.855
MT	ele_0–30°_	.479	.162	.139	.701	.602	.066	.345	.328
	ele_30–60°_	.430	.214	.200	.580	.456	.185	.273	.446
	ele_60–90°_	.527	.117	− .006	.987	.602	.066	.479	.162
	ele_90–120°_	.552	.098	.067	.855	.614	.059	.370	.293
	low_120–90°_	.188	.603	− .152	.676	.456	.185	− .042	.907
	low_90–60°_	.273	.446	− .091	.803	.535	.111	− .006	.987
	low_60–30°_	.309	.385	− .382	.276	.377	.283	− .164	.651
	low_30–0°_	− .018	.960	− .091	.803	.353	.318	− .091	.803
LT	ele_0–30°_	.503	.138	.164	.651	.511	.132	.370	.293
	ele_30–60°_	.576	.082	.079	.829	.559	.093	.455	.187
	ele_60–90°_	.564	.090	− .127	.726	.383	.275	.515	.128
	ele_90–120°_	.442	.200	.115	.751	.261	.466	.479	.162
	low_120–90°_	.370	.293	− .055	.881	.419	.228	.188	.603
	low_90–60°_	.479	.162	− .139	.701	.468	.172	.382	.276
	low_60–30°_	.503	.138	− .030	.934	.608	.062	.139	.701
	low_30–0°_	.176	.627	.006	.987	.474	.166	− .079	.829
SA	ele_0–30°_	.564	.090	.236	.511	.742*	**.014**	.273	.446
	ele_30–60°_	.442	.200	.261	.467	.596	.069	.224	.533
	ele_60–90°_	.491	.150	.224	.533	.657*	**.039**	.261	.467
	ele_90–120°_	.600	.067	.091	.803	.796*	**.006**	.491	.150
	low_120–90°_	.612	.060	.067	.855	.827*	**.003**	.115	.751
	low_90–60°_	.661*	**.038**	.042	.907	.881*	**.001**	.176	.627
	low_60–30°_	.600	.067	− .055	.881	.815*	**.004**	.055	.881
	low_30–0°_	.612	.060	.164	.651	.948*	**.000**	.418	.229
MD	ele_0–30°_	.042	.907	.067	.855	.219	.544	.394	.260
	ele_30–60°_	.079	.829	.030	.934	.146	.688	.430	.214
	ele_60–90°_	.030	.934	.139	.701	.061	.868	.430	.214
	ele_90–120°_	− .006	.987	.273	.446	.024	.947	.394	.260
	low_120–90°_	− .455	.187	.309	.385	− .146	.688	.503	.138
	low_90–60°_	− .455	.187	.382	.276	− .055	.881	.430	.214
	low_60–30°_	− .176	.627	.297	.405	.237	.510	.152	.676
	low_30–0°_	− .200	.580	.382	.276	.158	.663	.055	.881

*Significant correlation between the passive stiffness and the activity of periscapular muscles (*p* < 0.05).

*P* values set in bold indicate statistical significance.

### Relationship between muscle strength and scapular kinematics

Negative correlations were found in the maximal strength of UT (ele_30°_, r_s_ =  − 0.661, *p* = 0.038; ele_60°_, r_s_ =  − 0.636, *p* = 0.048) and SA (ele_30°_, r_s_ =  − 0.806, *p* = 0.005; ele_60°_, r_s_ =  − 0.636, *p* = 0.048) with regard to the internal rotations of the scapula. The maximal strength of UT (ele_30°_, r_s_ =  − 0.782, *p* = 0.001; ele_60°_, r_s_ =  − 0.806, *p* < 0.001; ele_90°_, r_s_ =  − 0.636, *p* = 0.05) also correlated negatively with the downward rotations of the scapula from ele_30°_ to ele_90°_ (Table 3), and the maximal strength of LT (ele_60°_, r_s_ =  − 0.709, *p* = 0.02; ele_90°_, r_s_ =  − 0.758, *p* = 0.02) (Table 3) and SA (ele_60°_, r_s_ =  − 0.648, *p* = 0.04; ele_90°_, r_s_ =  − 0.709, *p* = 0.02) (Table 3) negatively correlated with the downward rotations at ele_60°_ and ele_90°_ of scaption.

**Table 3:** The relationship between the maximal strength of periscapular muscles and the scapular kinematics during scaption.

Scapular kinematics		Muscle strength							
		UT		MT		LT		SA	
		r_s_	*p*	r_s_	*p*	r_s_	*p*	r_s_	*p*
Internal rotation	ele_30°_	− .661*	**.038**	− .479	.162	− .624	.054	− .806*	**.005**
	ele_60°_	− .636*	**.048**	− .418	.229	− .442	.200	− .636*	**.048**
	ele_90°_	− .273	.446	− .115	.751	− .042	.907	− .236	.511
	ele_120°_	− .212	.556	.006	.987	− .127	.726	− .309	.385
	low_120°_	− .236	.511	− .164	.651	− .612	.060	− .370	.293
	low_90°_	− .018	.960	.018	.960	− .236	.511	− .091	.803
	low_60°_	− .188	.603	− .018	.960	− .115	.751	− .212	.556
	low_30°_	− .115	.751	.067	.855	− .018	.960	− .139	.701
Downward rotation	ele_30°_	− .782*	**0.01**	− 0.44	0.20	− 0.58	0.08	− 0.58	0.08
	ele_60°_	− .806*	**0.00**	− 0.59	0.07	− .709*	**0.02**	− .648*	**0.04**
	ele_90°_	− .636*	**0.05**	− 0.38	0.28	− .758*	**0.01**	− .709*	**0.02**
	ele_120°_	− 0.19	0.60	− 0.04	0.91	− 0.54	0.11	− 0.54	0.11
	low_120°_	− .176	.627	− .030	.934	− .164	.651	− .055	.881
	low_90°_	− .103	.777	− .018	.960	− .224	.533	− .152	.676
	low_60°_	.164	.651	.103	.777	− .236	.511	− .188	.603
	low_30°_	.067	.855	.248	.489	− .261	.467	− .285	.425
Posterior tilting	ele_30°_	.164	.651	− .055	.881	.212	.556	.261	.467
	ele_60°_	− .006	.987	− .030	.934	.079	.829	− .018	.960
	ele_90°_	− .103	.777	− .139	.701	− .079	.829	− .200	.580
	ele_120°_	− .055	.881	− .030	.934	− .115	.751	− .309	.385
	low_120°_	.139	.701	.200	.580	.200	.580	.285	.425
	low_90°_	− .042	.907	.055	.881	− .006	.987	-.188	.603
	low_60°_	− .055	.881	.042	.907	− .042	.907	− .285	.425
	low_30°_	.091	.803	.042	.907	− .164	.651	− .236	.511

*Significant correlation between the maximal strength of periscapular muscles and the scapular kinematics (*p* < 0.05).

*P* values set in bold indicate statistical significance.

### Relationship between muscle strength and muscle activity

Our results showed that muscles with higher maximal strength had less sEMG activity during scaption. Negative correlations were found in UT (ele_0–30°_, r_s_ =  − 0.794, *p* = 0.06), MT (ele_0–30°_, r_s_ =  − 0.842, *p* = 0.02; ele_30–60°_, r_s_ =  − 0.830, *p* = 0.03; ele_60–90°_, r_s_ =  − 0.855, *p* = 0.02; ele_90–120°_, r_s_ =  − 0.709, *p* = 0.02) and LT (ele_0–30°_, r_s_ =  − 0.661, *p* = 0.038) but not in SA. Muscle strength not only affected the activity of the muscle in question, but also negatively correlated with the activity of adjacent muscles. The maximal strength of UT negatively correlated with the activity of MD (ele_60–90°_, r_s_ =  − 0.661, *p* = 0.038; ele_90–120°_, r_s_ =  − 0.782, *p* = 0.008), MT (ele_0–30°_, r_s_ =  − 0.806, *p* = 0.05; ele_30–60°_, r_s_ =  − 0.842, *p* = 0.02; ele_60–90°_, r_s_ =  − 0.903, *p* < 0.001; ele_90–120°_, r_s_ =  − 0.867, *p* = 0.01) and LT (ele_0–30°_, r_s_ =  − 0.770, *p* = 0.009; ele_30–60°_, r_s_ =  − 0.855, *p* = 0.002; ele_60–90°_, r_s_ =  − 0.745, *p* = 0.013; ele_90–120°_, r_s_ =  − 0.818, *p* = 0.004) (Table 4). Similar patterns were observed in MT, where the maximal strength negatively correlated with the activity of MD (ele_30–60°_, r_s_ =  − 0.636, *p* = 0.048; ele_60–90°_, r_s_ =  − 0.636, *p* = 0.048) and LT (ele_0–30°_, r_s_ =  − 0.867, *p* = 0.001; ele_30–60°_, r_s_ =  − 0.915, *p* < 0.001; ele_60–90°_, r_s_ =  − 0.782, *p* = 0.008; ele_90–120°_, r_s_ =  − 0.709, *p* = 0.022) (Table 4); in LT, where the maximal strength negatively correlated with the activity of MD (ele_90–120°_, r_s_ =  − 0.673, *p* = 0.033) and MT (ele_30–60°_, r_s_ =  − 0.685, *p* = 0.029; ele_90–120°_, r_s_ =  − 0.648, *p* = 0.043) (Table 4); and in SA, where the maximal strength negatively correlated with the activity of MD (ele_30–60°_, r_s_ =  − 0.667, *p* = 0.038; ele_60–90°_, r_s_ =  − 0.721, *p* = 0.017; ele_90–120°_, r_s_ =  − 0.745, *p* = 0.013) and MT (ele_30–60°_, r_s_ =  − 0.661, *p* = 0.038) (Table 4).

**Table 4:** The relationship between the maximal strength and the muscle activity of periscapular muscle during scaption.

Muscle activity		Muscle strength							
		UT		MT		LT		SA	
		r_s_	*p*	r_s_	*p*	r_s_	*p*	r_s_	*p*
UT	ele_0–30°_	− .055	.881	− .285	.425	− .188	.603	.297	.405
	ele_30–60°_	− .224	.533	− .370	.293	− .333	.347	.212	.556
	ele_60–90°_	− .455	.187	− .503	.138	− .539	.108	− .055	.881
	ele_90–120°_	− .794*	**.006**	− .600	.067	− .491	.150	− .333	.347
	low_120–90°_	− .212	.556	− .006	.987	.212	.556	.370	.293
	low_90–60°_	− .127	.726	.079	.829	.115	.751	.418	.229
	low_60–30°_	.333	.347	.333	.347	.248	.489	.576	.082
	low_30–0°_	.406	.244	.273	.446	.685*	**.029**	.673*	**.033**
MT	ele_0–30°_	− .806*	**.005**	− .842*	**.002**	− .491	.150	− .455	.187
	ele_30–60°_	− .842*	**.002**	− .830*	**.003**	− .685*	**.029**	− .661*	**.038**
	ele_60–90°_	− .903*	**.000**	− .855*	**.002**	− .467	.174	− .515	.128
	ele_90–120°_	− .867*	**.001**	− .709*	**.022**	− .648*	**.043**	− .576	.082
	low_120–90°_	− .212	.556	− .200	.580	.176	.627	.006	.987
	low_90–60°_	− .261	.467	− .224	.533	.127	.726	− .006	.987
	low_60–30°_	− .164	.651	− .188	.603	.236	.511	− .067	.855
	low_30–0°_	− .030	.934	− .067	.855	.248	.489	.030	.934
LT	ele_0–30°_	− .770*	**.009**	− .867*	**.001**	− .661*	**.038**	− .479	.162
	ele_30–60°_	− .855*	**.002**	− .915*	**.000**	− .588	.074	− .430	.214
	ele_60–90°_	− .745*	**.013**	− .782*	**.008**	− .564	.090	− .479	.162
	ele_90–120°_	− .818*	**.004**	− .709*	**.022**	− .406	.244	− .321	.365
	low_120–90°_	− .406	.244	− .442	.200	.030	.934	− .127	.726
	low_90–60°_	− .588	.074	− .661*	**.038**	− .042	.907	− .236	.511
	low_60–30°_	− .406	.244	− .479	.162	.139	.701	− .018	.960
	low_30–0°_	− .176	.627	− .261	.467	.139	.701	− .006	.987
SA	ele_0–30°_	− .455	.187	− .576	.082	− .285	.425	.055	.881
	ele_30–60°_	− .467	.174	− .612	.060	− .467	.174	− .139	.701
	ele_60–90°_	− .491	.150	− .624	.054	− .406	.244	− .067	.855
	ele_90–120°_	− .661*	**.038**	− .745*	**.013**	− .370	.293	− .164	.651
	low_120–90°_	− .200	.580	− .248	.489	.200	.580	.139	.701
	low_90–60°_	− .152	.676	− .248	.489	.055	.881	.139	.701
	low_60–30°_	− .067	.855	− .188	.603	− .006	.987	.103	.777
	low_30–0°_	− .455	.187	− .491	.150	.006	.987	.091	.803
MD	ele_0–30°_	− .491	.150	− .552	.098	− .527	.117	− .564	.090
	ele_30–60°_	− .624	.054	− .636*	**.048**	− .576	.082	− .661*	**.038**
	ele_60–90°_	− .661*	**.038**	− .636*	**.048**	− .612	.060	− .721*	**.019**
	ele_90–120°_	− .648*	**.043**	− .564	.090	− .673*	**.033**	− .745*	**.013**
	low_120–90°_	− .152	.676	− .164	.651	− .188	.603	− .309	.385
	low_90–60°_	− .273	.446	− .115	.751	.006	.987	− .358	.310
	low_60–30°_	− .115	.751	− .127	.726	− .103	.777	− .248	.489
	low_30–0°_	− .055	.881	− .006	.987	.127	.726	− .139	.701

*Significant correlation between the maximal strength and the muscle activity of periscapular muscle (*p* < 0.05).

*P* values set in bold indicate statistical significance.

## Discussion

In the present study, swimmers experiencing increased stiffness of UT and MT showed decreases in upward rotation and increases in internal rotation of the scapula, respectively. Higher maximal strength of UT, LT, and SA correlated with the range of upward rotation during scaption. Increases in the strength of UT and SA were associated with a decrease in internal rotation of the scapula. Negative correlations between the maximal strength and its activities during scaption were found in UT, MT, and LT. In addition, the activities of MD during scaption negatively correlated with the strength of periscapular muscles.

Previous studies have shown the adaptive changes in strength of the periscapular muscles in swimmers after swim training, which may be associated with SD and altered neuromuscular control. There were more competitive swimmers exhibiting SD due to muscle fatigue after swim training than before training^25,26^ Swimmers also demonstrate a decrease in SA activity and an increase in internal rotation and anterior tilting of the scapula during scaption after maximal-effort stroke exercise^27^ The possible reason was SA fatigue since SA alternately played as a scapular stabilizer and protractor throughout the swimming stroke. However, more internal rotation and anterior tilting of the scapula with higher activity of the SA during scaption were noticed in swimmers than in nonpractitioners^12^ The increases in SA activity didn’t favor tilting the scapula posteriorly and rotating it externally may be due to insufficient strength. A longitudinal study also found decreased strength in the shoulder external rotator, supraspinatus, and lower trapezius after three years of swim training in adolescent swimmers^12^.

Higher maximal strength of the periscapular muscles was associated with increases in upward rotation and external rotation of the scapula. The maximal strength of MT, SA, and UT were accompanied with the upward rotation of the scapula in overhead athletes^11,25^. Although SD is not sensitive nor specific to shoulder disorders, the decreased upward rotation of the scapula was noticed in patients with subacromial impingement syndrome or shoulder instability^11,25^ It was reported that the activities of SA, MT, and LT had to be elevated to increase the upward rotation and posterior tilting of the scapula as compensation for the insufficient subacromial space^28^ However, some contrasting findings were noticed, where there was no significant difference in the strength of periscapular muscles between the patients with and without SD^23^ Other studies also found improvements in self-reported pain status and functions after scapula-focused training, but patients elevated their arms with unchanged scapulohumeral movements^29,30^ As for healthy swimmers, the role of scapular mechanics in kinetic chain of upper extremity should be integrated into the training protocol^31^ Future studies will be needed to evaluate the benefits on increased efficiency of stroke propulsion during front-crawl swimming after strengthening the periscapular muscles.

In the current study, the effects of of periscapular muscle stiffness on scapular neuromuscular controls were observed during the lowering phase of scaption. A previous study reported that the stiffness of the UT and MT developed due to muscle lengthening by long-term scapular depression and downward rotation^32^ In contrast, the deceleration phase during overhead sports requires eccentric contractions by the periscapular and posterior shoulder muscles. Stiffness in UT^33^ or LT^18^ may develop rapidly after overhead sports training. The volleyball players with rotator cuff tendinopathy showed higher stiffness in UT than the asymptomatic players^33^ The increased stiffness in UT may be due to the consequence of compensation by increasing the activation level of UT for shoulder movement and keeping a forward shoulder posture in players with tendinopathy. Increases in stiffness of the LT and infraspinatus muscles were noticed in healthy baseball pitchers after one hundred pitches and sustained for one day^18^ The sustained stiffness of these two muscles may be induced by enhanced activations during the deceleration phase of pitching to stabilize the scapula and decelerate the humerus. In the present study, the downward and internal rotations of scapula may have lengthened the muscle and affected the length-tension relationship of the UT and MT, consequently causing muscle stiffness. It was also noted that muscle stiffness affected the activities of other periscapular muscles. The increased UT activity may possibly have compensated for the stiff SA, and the increased activity of SA for the stiffness of LT. This may imply that UT and SA serve as the compensators for the stiffness of other periscapular muscles to preserve the scapular rotations. The UT, LT, and SA are important members of these force couples, and they work together to rotate the scapula^6,10,34^.

Reduced muscle activity during scaption were noted in the UT, MT, and LT with higher values of maximal strength than in those with lower values. It was reported that rectus femoris with higher values of maximal strength had lower levels of muscle activity during a knee extension task than those with lower values of maximal strength^35,36^ This relationship can also be validated by the finding that the recruitment threshold of motor units in the tibialis anterior decreased in parallel with increased maximal strength after training^37^ Although strengthening training of the periscapular muscles is commonly prescribed for patients with shoulder disorders^29,31^, no studies have investigated the relationship between the strength capacity and muscle activity of the periscapular muscles. Future studies need to compare the effects of increases in periscapular muscle strength on its neuromuscular controls and scapular kinematics in swimmers.

Negative correlations were found between the maximal strength of periscapular muscles (UT, MT, LT, and SA) and the muscle activity of the MD during scaption. MD may increase its contribution to compensate for the poor control of the scapula during arm elevation. Higher MD activity was noticed in protracted shoulder posture than in neutral shoulder posture due to the altered length-tension relationship^38^ Over-activated MD during arm elevation was exhibited in patients with rotator cuff tears^39,40^ Fatigue in an over-activated MD may thus develop after repetitive and forceful training. Thus, stiffness or fatigue symptoms of MD should be monitored in swimmers with imbalanced strength in the periscapular muscles. The relationship of strength of periscapular muscles with regard to deltoids activity during shoulder movement requires further investigation to provide a reference for optimizing the strength balance of athletes.

### Limitation

The present results may only be generalized to the competitive adolescent swimmers practicing middle- and long-distance with front-crawl stroke. Swimmers using other stroke styles possibly present different patterns of stiffness and strength of periscapular muscles. In addition, these correlation results can not imply the causation among variables.

## Conclusion

This observational study found that the passive stiffness and maximal strength of periscapular muscles were associated with muscle activity and shoulder kinematics during the elevation or lowering phase of scaption in competitive adolescent swimmers. The training program for competitive swimmers with SD may consider the strength and stiffness of periscapular muscles in addition to the scapular kinematics. Future studies should investigate the effects of strengthening for imbalanced strength among periscapular muscles or relaxation for stiff muscles on scapular kinematics and neuromuscular controls in competitive swimmers.

## Supplementary Information

Supplementary Information 1.

Supplementary Information 2.

## Data Availability

The datasets analyzed in this study are available from the corresponding author upon reasonable request.
