# Supplementary material for: Scapulohumeral kinematics and neuromuscular control during scaption are associated with passive stiffness and strength of periscapular muscles in competitive adolescent swimmers
Source: Sci Rep. 2023 Jan 13;13:725. doi: 10.1038/s41598-023-27920-w (PMC9839700; doi:10.1038/s41598-023-27920-w)
Supplement: Supplementary file 1 — Supplementary Information 1. [file 41598_2023_27920_MOESM1_ESM.docx]

Legend of Tables

UT: upper trapezius

MT: middle trapezius

LT: lower trapezius

SA: serratus anterior

MD: middle deltoid

stiff: stiffness

strength: maximal strength

IR: internal rotation of scapula

PT: posterior tilting of scapula

DR: downward rotation of scapula

ele30, ele60, ele90, ele120: 30°, 60°, 90°, and 120° of elevation phase during scaption, respectively

low30, low60, low90, low120: 30°, 60°, 90°, and 120° of lowering phase during scaption, respectively

ele0-30, ele30-60, ele60-90, ele90-120: range of 0-30°, 30-60°, 60-90°, and 90-120° of elevation phase during scaption, respectively

low0-30, low 30-60, low60-90, low90-120: range of 0-30°, 30-60°, 60-90°, and 90-120° of lowering phase during scaption, respectively

stiff_UT, stiff_MT, stiff_LT and stiff_SA: stiffness of UT, MT, LT, and SA, respectively

strength_UT, strength_MD, strength_MT, strength_LT and strength_SA: maximal strength of UT, MD, MT, LT, and SA, respectively

IR_ele30: internal rotation of scapula at elevation 30°

IR_low30: internal rotation of scapula at lowering 30°

PT_ele30: posterior tilting of scapula at elevation 30°

PT_low30: posterior tilting of scapula at lowering 30°

DR_ele30: downward rotation of scapula at elevation 30°

DR_low30: downward rotation of scapula at lowering 30°

UT_ele0-30: mean activity of UT during elevation 0-30°

UT_low0-30: mean activity of UT during lowering 0-30°

SA_ele0-30: mean activity of SA during elevation 0-30°

SA_low0-30: mean activity of SA during lowering 0-30°

MD_ele0-30: mean activity of SA during elevation 0-30°

MD_low0-30: mean activity of SA during lowering 0-30°

LT_ele0-30: mean activity of LT during elevation 0-30°

LT_low0-30: mean activity of LT during lowering 0-30°

MT_ele0-30: mean activity of MT during elevation 0-30°

MT_low0-30: mean activity of MT during lowering 0-30°
